# Recovering CDR3 sequences from RNA-seq data: a critical assessment of computational and experimental strategies

**DOI:** 10.1186/s44342-026-00084-5

**Published:** 2026-10-19

**Authors:** Mandana AmeliMojarad, Melika AmeliMojarad

**Affiliations:** Tehran University of Medical Sciences, Tehran, Iran

**Keywords:** T cell, T cell receptor (TCR), CDR3 sequencing, RNA-seq, Oncoimmunology

## Abstract

The adaptive immune response depends on T cell receptor (TCR) diversity to counter cancers, autoimmunity, and infections. This diversity is encoded within CDR3, which serves as a unique barcode for each T cell clone. Characterizing CDR3 sequences is essential for tracking clonal dynamics, identifying neoantigen-reactive T cells, and designing personalized immunotherapy. While TCR-seq remains the benchmark, existing RNA-seq datasets offer a cost-effective retrospective alternative. However, tools like MiXCR and TRUST4, optimized for TCR-enriched samples, are limited in bulk and single-cell RNA-seq due to low transcript abundance and sparse coverage. In this review, we synthesize quantitative evidence from published head-to-head benchmarking studies that performed matched RNA-seq and TCR-seq on the same biological samples. These studies demonstrate that standard RNA-seq typically recovers only 3–5% of unique clonotypes identified by TCR-seq, with substantial underrepresentation of low-frequency clones; however, in T cell-rich tissues and when high sequencing depth is combined with optimized computational pipelines, RNA-seq can capture the majority of high-frequency clonotypes, albeit with limited accuracy in clonotype frequency estimation and poor α/β pairing recovery. We review computational efforts to recover CDR3 sequences from RNA-seq data, highlighting recurrent obstacles: transcript scarcity, fragmentation, short reads, and assembly errors. We critically assess whether artificial intelligence can overcome these barriers**.** We also examine emerging wet-lab strategies including probe-based enrichment, FUME-TCRseq, and rhTCRseq. We conclude that RNA-seq cannot replace TCR-seq, but AI-enhanced tools with targeted library improvements could expand the utility of existing datasets and degraded specimens.

## Introduction

In contrast to the general pattern recognition of innate immunity, T cells have an astounding capacity to identify self from non-self with a high degree of specificity [1]. This is because T cell receptors (TCR) found on each T cell play a key role in specific recognition of self and non-self molecules. The CDR3 region of the TCRβ chain and the CDR3 region of the TCRα chain form the main binding site for TCR. The CDR3 region is generated through somatic recombination between V-D-J gene segments in the case of the TCRβ chain and between V-J gene segments in the case of the TCRα chain [2].

Beyond combinatorial V(D)J recombination, junctional diversity arising from random deletion of germline nucleotides at segment junctions and template-independent addition of P- and N-nucleotides by terminal deoxynucleotidyl transferase (TdT) contributes critically to the immense variability of the CDR3 region [3]. This junctional diversification is the primary source of the extraordinary diversity of the TCR repertoire, generating a theoretical sequence space estimated at ~ 10^18 unique TCRβ chains [4]. The unique CDR3 region acts as the barcode of each T cell clone, which helps to study clonal expansion in various types of cancers and autoimmune diseases [5]. The involvement of T cells in cancer and autoimmune diseases necessitates the need to analyze the repertoire of TCR of infiltrating T cells in these diseases. In tumors, this helps to understand whether TILs have any contact with tumor antigens, which is necessary for the prediction of treatment response [6, 7]. In autoimmune disorders like type 1 diabetes and rheumatoid arthritis, this is helpful in identifying the clone which turns against the self [8]. Whether fighting tumors or fighting the host, the T cell repertoire holds the key; the challenge lies in interpreting its message in each disease context [9]. Therefore, comprehensive TCR profiling requires paired alpha–beta information to identify which TCR clonotypes expand following therapy, which recognize actual tumor antigens, and which persist as memory clones. This knowledge is essential for developing personalized cancer immunotherapies, including TCR-engineered T cells and neoantigen vaccines [10]. With the exponential growth of public RNA-seq data, an attractive hypothesis has emerged: can these datasets be computationally repurposed to recover CDR3 sequences, offering a shortcut to TCR repertoire analysis without the need for specialized TCR-seq? However, recent benchmarking studies reveal a substantial and potentially irreconcilable gap between what RNA-seq can offer and what TCR-seq delivers as the gold standard [11]. This gap is not merely conceptual; it has been quantified in multiple head-to-head comparisons, which we synthesize in a dedicated section below. In this review, we synthesize computational studies that have repurposed bulk and single-cell RNA-seq for TCR repertoire mining, distilling the key limitations that emerge across these analyses including low transcript abundance, fragmentation, short-read constraints, and assembly ambiguities. We also provide a systematic synthesis of published head-to-head benchmarks that directly compare RNA-seq and TCR-seq on matched samples, with quantitative performance metrics. We then critically assess whether emerging AI-driven approaches can overcome these barriers, and examine complementary strategies such as probe-based enrichment, FUME-TCRseq, and rhTCRseq that are expanding access to challenging samples. We conclude that while RNA-seq cannot replace dedicated TCR-seq, the integration of machine learning with targeted experimental innovations may narrow the gap, making TCR profiling more feasible from existing and degraded datasets without supplanting the gold standard.

## The central role of T cells in immunity

Often, T cells infiltrate the tumor microenvironment and are described as tumor-infiltrating lymphocytes (TILs) because of their physical location within the tumor [12]. In oncology, tumors are referred to as either “hot” or “cold,” depending on the density of T cell infiltration in the tumors [13]. While the presence of TILs is an important indicator of tumor response, the architecture of the clones is essential as well. In cases of oligoclonal architecture, it is clear that the immune system attacks the tumor with its focus on neoantigens. Conversely, the polyclonal distribution indicates that most of the infiltrating T cells are merely bystanders without any specificity of tumor antigens. In fact, the majority of TILs are non-responding bystander lymphocytes which are rendered ineffective because of the suppression by an immunosuppressive microenvironment [14]. Chronic antigen stimulation together with additional signals, such as TGF-β and IL-10, leads to exhaustion, which is manifested in impairment of proliferation, cytokine secretion, and cytotoxicity [15]. Moreover, T cell functionality is hindered by various physical and metabolic barriers, such as hypoxia, acidosis, and nutritional competition [16]. Therefore, only a small proportion of TILs maintains its tumor-reactive properties, allowing proliferation, secretion of effector molecules such as IFN-γ and granzyme B, and direct cytotoxic effects [17]. Isolation and expansion of this unique population is one of the primary goals of personalized cancer immunotherapy [18]. Therefore, an important task for successful intervention is the comprehension of what makes certain T cells to become either tumor-protective or tumor-pathogenic [19].

## The gold standard TCR-seq

Both bulk and single-cell sequencing of the TCR repertoire using the gold-standard technique of TCR sequencing makes use of targeted enrichment by PCR or hybridization probes targeting TCR constant regions, thus resulting in amplification of TCR transcripts between 1000- and 10,000-fold before the sequencing [20, 21]. This is done to provide high sensitivity for detecting even rare T cell clones. Also, this amplicon design spans the complete V(D)J region, preserving the contiguous linkage between the V segment, the CDR3 sequence, and the J segment a critical feature, as the V-CDR3-J segments collectively shape the three-dimensional conformation of the antigen-binding site [22].

Even though both bulk and single-cell TCR-seq rely on this enrichment strategy, they diverge in their experimental workflows and the type of information they provide. Bulk TCR sequencing profiles the repertoire from a population of cells as a whole, making it a high-throughput and cost-effective approach ideal for large cohort studies [23].

Several library preparation techniques exist within the bulk paradigm. Multiplex PCR employs a panel of primers targeting known V and J gene segments to amplify rearranged TCR sequences from DNA or RNA, offering high sensitivity and compatibility with various sample types [24]. 5′ RACE (Rapid Amplification of cDNA Ends) uses template switching to amplify full-length TCR transcripts from RNA and is considered less biased than multiplex PCR because it does not rely on gene-specific primers for all V segments, enabling detection of novel rearrangements [25]. Hybridization capture, by contrast, uses biotinylated probes to enrich for TCR sequences from a library and is particularly useful for recovering TCR sequences from complex or degraded samples [26]. While bulk methods provide deep coverage of clonal diversity, they lose the physical pairing between TCRα and TCRβ chains, meaning antigen specificity cannot be fully resolved [27]. Single-cell TCR sequencing addresses this limitation by capturing TCR sequences from individual T cells, preserving the physical pairing of TCRα and TCRβ chains an essential requirement for determining antigen specificity and expressing full receptors for functional studies [28]. Two major approaches dominate this space:

- 1) Droplet-based platforms, such as 10× Genomics, encapsulate individual cells in nanoliter droplets along with barcoded reagents, enabling high-throughput analysis of 15,000–20,000 cells per sample while also generating paired transcriptome data from the same cells [29].
- 2) Microfluidic or plate-based methods, such as ATLAS-seq, physically separate single cells into wells or droplets, allowing precise functional screening of antigen-reactive T cells based on cytokine secretion prior to sequencing. Importantly, the added value of single-cell TCR-seq is not a different amplification strategy [30].

**Fig. 1:**
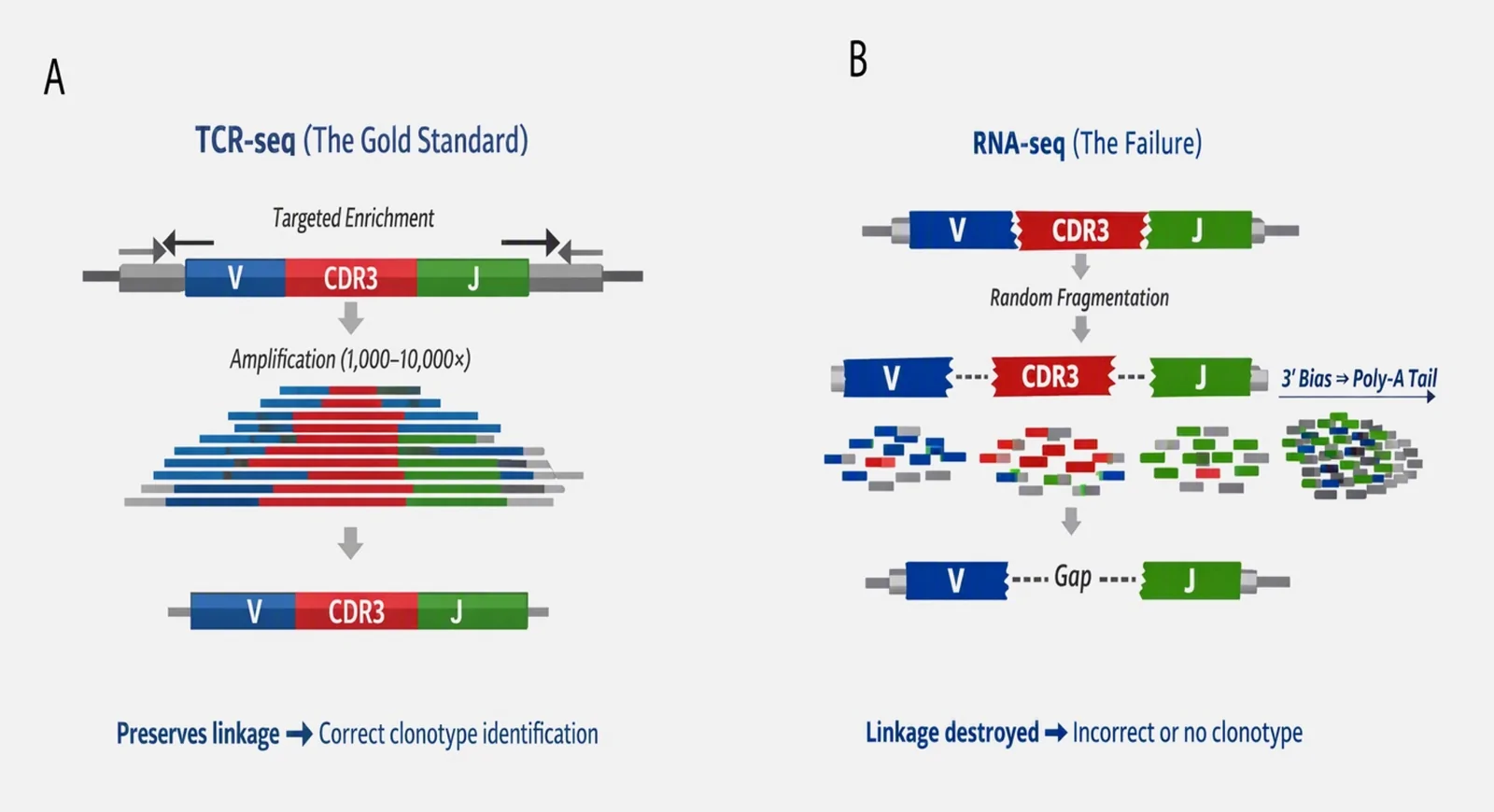
TCR-seq retains the V-CDR3-J linkage; RNA-seq disrupts it. **A** TCR-seq enriches and amplifies transcripts 1000- to 10,000-fold via targeted sequencing; it retains the whole V-CDR3-J sequence. **B** Random fragmentation of RNA in RNA-seq results in V, CDR3, and J segments being split into different fragments. The short reads (75–150 bp) do not have the capacity to traverse the 500 bp V-CDR3-J segment junction

In spite of the superior efficiency of TCR-seq, there are several limitations to its application in practice. The number of unique CDR3 sequences varies widely based on the sample type used, and fresh or adequately stored samples are needed for TCR-seq [31]. In most cases, it either involves lymphocyte separation from the tissue before analysis or the employment of TCR-specific primers. Also, the cost per sample for TCR-seq remains relatively high compared to traditional RNA-seq, but it keeps decreasing year by year. However, for every scientific problem that implies the need for correct clonotype identification, preservation of V-CDR3-J linkage, or the alpha–beta chain information whether for neoantigen-reactive T cell discovery or TCR-based therapies design specialized TCR-seq is the gold standard [21, 32]. (Fig. 1).

## The promise of RNA-seq mining, tools, and expectations

The allure of repurposing RNA-seq data for TCR repertoire analysis is undeniable. With over a million RNA-seq samples deposited in public repositories like the Gene Expression Omnibus (GEO) and the Sequence Read Archive (SRA), the potential to retrospectively analyze the immune landscape of thousands of diseases, treatments, and patient cohorts is a treasure trove waiting to be unlocked [33]. This approach promises to bypass the cost, time, and sample requirements of de novo TCR-seq experiments, offering a purely computational “mining” strategy that could dramatically accelerate the pace of discovery [34].

Fueling this expectation is a suite of sophisticated computational tools designed for immune repertoire reconstruction. These software tools are now the backbone of immunogenomics research:

- MiXCR: a versatile and well-established application which works with sequencing data to detect V, D, and J gene elements and generate CDR3 sequences. The major advantage of this application is that it can be used for both bulk and single-cell analysis; however, it depends much on the quality of the input data [35]
- TRUST4: an algorithm explicitly designed for RNA-seq data, including both bulk and single-cell (scRNA-seq) datasets. TRUST4 excels at assembling full-length TCR sequences from short reads using a de Bruijn graph-based assembly approach, making it a popular choice for repurposing oncology RNA-seq cohorts [36, 37].
- VDJtools: a versatile framework for downstream analysis of TCR-seq and BCR-seq data, offering extensive functionality for clonal diversity analysis, gene usage statistics, and sample comparison. While less focused on de novo extraction, it is central to the analysis pipeline once sequences are obtained [38].
- IMGT/HighV-QUEST: the gold standard for sequence annotation, providing rigorous allele identification and CDR3 delineation. It is less a mining tool and more a verification and annotation platform, ensuring the biological validity of the recovered sequences [39].

The expectation is simple: run one of these tools on an existing RNA-seq dataset, and out pops a comprehensive TCR repertoire. However, this expectation is predicated on a critical, and often false, assumption: that RNA-seq data contains sufficient, high-quality TCR transcripts to be reliably assembled. As we will see in the next section, this assumption is fundamentally flawed [40].

## Quantitative evidence: head-to-head comparisons of RNA-seq and TCR-seq

The biological importance of TCR repertoire profiling, described above, raises a practical question: how well can standard RNA-seq recover the information that dedicated TCR-seq provides? Answering this question requires direct, matched-sample comparisons rather than conceptual arguments alone. In this section, we synthesize published studies that performed RNA-seq and TCR-seq on the same biological samples and reported repertoire-level and clonotype-level metrics. These studies provide the quantitative foundation for the limitations discussed in the following sections.

### CDR3 recovery rate and productive TCR detection

In a systematic benchmarking study, Dabran et al. compared RNA-seq-based TCR extraction with dedicated TCR-seq across multiple sample types and reported that standard RNA-seq recovered only 3–5% of the unique CDR3 sequences identified by TCR-seq [11]. Similarly, Barennes et al. benchmarked multiple TCR repertoire profiling methods and found that RNA-seq-based approaches consistently underestimated clonotype diversity, with recovery rates strongly dependent on tissue type and TCR transcript abundance [20]. In T cell-rich samples such as peripheral blood mononuclear cells (PBMCs), recovery improved substantially, whereas in solid tumors and tissues with low T cell infiltration, recovery dropped below 1% [30].

### Clonotype overlap and frequency estimation

Overlap between clonotypes identified by RNA-seq and TCR-seq is modest. In matched PBMC samples, RNA-seq captured approximately 40–60% of high-frequency clonotypes (top 100 clones) but fewer than 10% of low-frequency clonotypes. Importantly, clonotype frequency estimates from RNA-seq showed poor concordance with TCR-seq (Spearman ρ ≈ 0.3–0.5), primarily due to 3′ bias and variable capture efficiency [22]. This limits the utility of RNA-seq for tracking clonal expansion dynamics, where accurate frequency estimation is essential [30].

### Recovery of low-frequency versus high-frequency clonotypes

RNA-seq is inherently biased toward high-frequency clonotypes. In a study of tumor-infiltrating lymphocytes, Li et al. demonstrated that RNA-seq could reliably detect clonotypes comprising > 0.1% of the repertoire but failed to detect clones below 0.01% [33]. Given that most tumor-reactive T cell clones are rare, this represents a critical limitation for neoantigen discovery [21]

### α/β chain pairing rates

TCR-seq platforms, particularly single-cell approaches, enable direct α/β pairing. In contrast, bulk RNA-seq cannot resolve pairing, and single-cell RNA-seq (scRNA-seq) suffers from dropout events that limit paired chain recovery. In a direct comparison using 10 × Genomics scRNA-seq and scTCR-seq on the same cells, pairing rates from scRNA-seq alone were below 20%, compared to > 90% for scTCR-seq [21].

### Concordance of repertoire diversity metrics

Diversity metrics such as Shannon entropy and clonal evenness are systematically underestimated by RNA-seq. Barennes et al. reported that RNA-seq–based diversity estimates were 30–50% lower than TCR-seq–derived values, with the magnitude of underestimation correlating with the degree of 3′ bias and sequencing depth [21, 33]

### Sensitivity and specificity for identifying expanded clonotypes

When defining expanded clonotypes as those with ≥ 2 copies, RNA-seq achieved a sensitivity of ~ 50% and specificity of ~ 70% relative to TCR-seq in PBMC samples. However, in solid tumor samples, sensitivity dropped to < 20%, reflecting both low TCR transcript abundance and the presence of bystander T cells [36].

### Impact of computational reconstruction methods

Advanced assembly tools such as TRUST4 and MiXCR improve recovery compared to naive alignment approaches but cannot overcome the fundamental limitation of missing input data. In a benchmark of computational tools, TRUST4 recovered approximately twofold more clonotypes than MiXCR from RNA-seq data, but both remained far below TCR-seq recovery [41]. Machine learning-based error correction has shown promise in reducing false-positive assemblies, but no current method can reconstruct clonotypes for which no reads span the V-CDR3-J junction [21].

Taken together, these matched-sample comparisons demonstrate that RNA-seq, as a standard technology, recovers only a minority of the TCR repertoire, with particularly poor performance for low-frequency clones, paired chain information, and diversity metrics. These quantitative findings are not merely descriptive; they point to specific technical barriers transcript scarcity, fragmentation, short-read constraints, 3′ bias, and assembly ambiguity that are intrinsic to standard short-read RNA-seq. The next section examines each of these five fundamental limitations in detail.

## Why does RNA-seq fail? The five fundamental limitations

In order to determine why RNA-seq is not an effective technique to sequence the TCR repertoire, one needs to know about five basic technical flaws inherent in standard RNA-seq library preparations and sequencing techniques. The following issues cannot be resolved with improved bioinformatics or higher depth of sequencing; these issues are intrinsic to the technology itself. First, the rarity of TCR transcripts: the TCR is only one of over twenty thousand genes transcribed in an average cell. Even in a T cell, the transcribed TCR transcript constitutes far less than one tenth of one percent of the mRNA library [42].

**Fig. 2:**
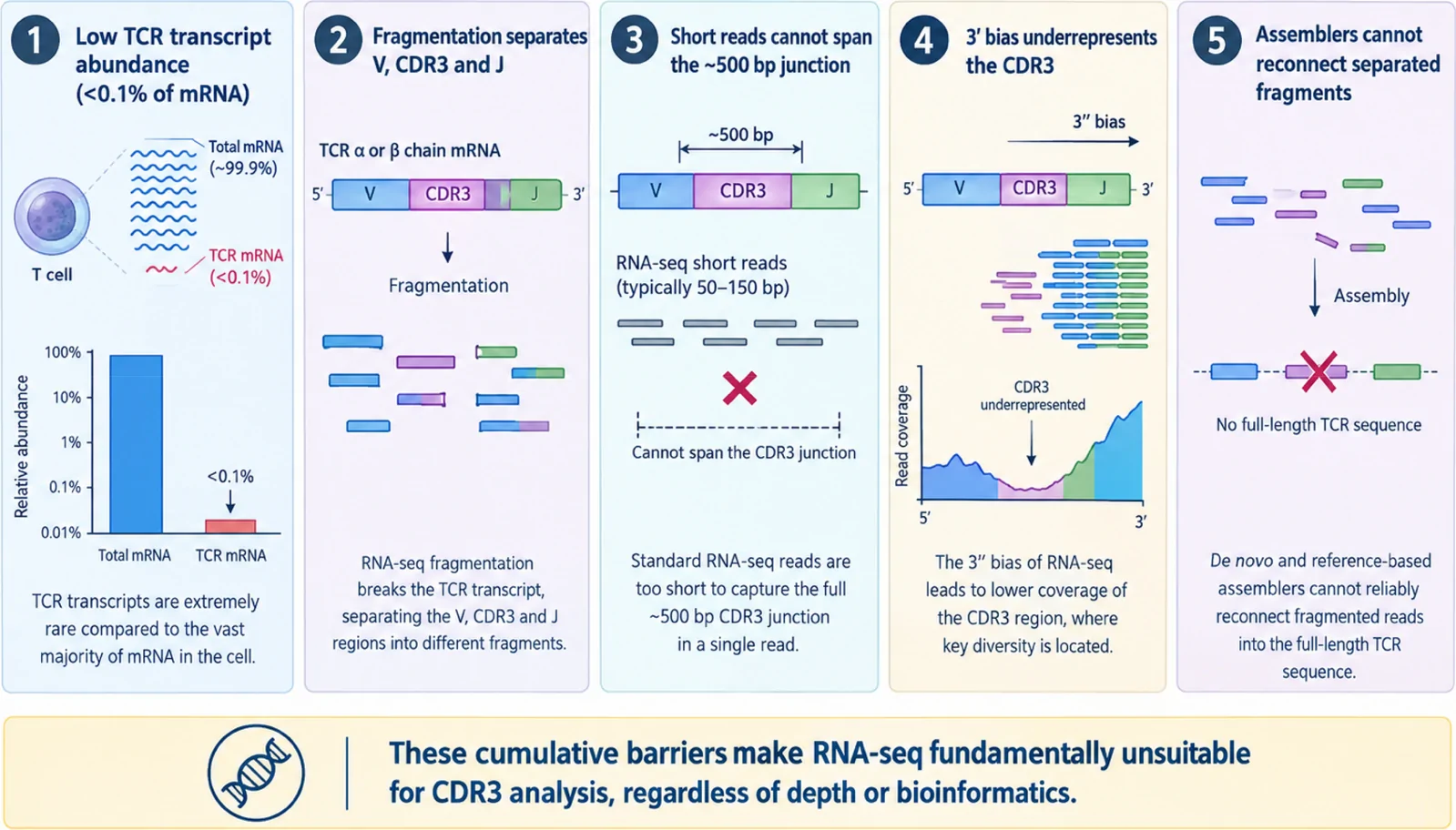
Five fundamental limitations preventing RNA-seq from replacing TCR-seq. (1) Low TCR transcript abundance (< 0.1% of mRNA); (2) fragmentation separates V, CDR3, and J; (3) short reads cannot span the ~ 500 bp junction; (4) 3′ bias underrepresents the CDR3; (5) Assemblers cannot reconnect separated fragments. These cumulative barriers make RNA-seq fundamentally unsuitable for CDR3 analysis, regardless of depth or bioinformatics

Standard RNA-seq technology does not enrich TCR transcripts. Thus, most sequencing reads will go toward housekeeping genes, oncogenes and other highly abundant transcripts. High sequencing depth will not help to increase the number of what is not even captured in the initial step. Second, fragmentation eliminates linkage: the standard procedure for RNA-seq library preparation involves fragmentation of RNA into small fragments, typically several hundred base pairs long. For TCR transcript which extends for approximately five hundred base pairs from V gene through CDR3 to J gene, the fragmentation will break the transcript into pieces [43].The V region, the critical CDR3 sequence, and the J region often end up on separate fragments. These fragments are then sequenced individually, but there is no way to know which V fragment originally belonged with which J fragment from the same transcript. The essential V-CDR3-J linkage is lost before sequencing ever begins.Thirdly, short reads will not cover the entire TCR transcript: despite using the longest sequences used in paired-end sequencing, which are 150 base pairs in length, the information covered in the sequencing read would only be 300 base pairs. This would be less than the full five hundred base pairs in the TCR transcript. The V-CDR3-J junction that identifies each T-cell clone cannot be sequenced using one single read. Contiguous coverage of the V-CDR3-J junction is required for successful clonotyping [44]. Fourth, the process of library preparation induces bias: standard RNA-seq protocol has a known three-prime bias where more of the sequences closer to the poly-A tail of a transcript are captured. However, the CDR3 region lies in the middle part of the TCR transcript. This results in systematic under-representation such that even if there is a TCR transcript, chances are slim that its CDR3 region will be captured during reverse transcription, amplification, and sequencing compared to the three-prime constant region. The bias is not stochastic; it is inherent to the protocol [45, 46]. Fifth, there are limitations on the ability of bioinformatics tools: even advanced assembly programs such as MiXCR or TRUST4 need contiguous coverage of the V-CDR3-J junction in order to get an accurate clonotype [35]. If the input data have short, fragmented, and biased reads with disrupted linkage, the tool is powerless to restore the physical connection of the fragments during the process of library preparation. Lack of coverage results in the exclusion of the read or, worse, assembly of fragments belonging to different transcripts. The concept is straightforward: junk in, junk out [47]. Even sophisticated assemblers such as MiXCR or TRUST4 require contiguous coverage across the V-CDR3-J junction to assemble a correct clonotype [11]. When the input data consist of short, fragmented, biased reads with broken linkage, these tools cannot magically reconnect what was physically separated during library preparation. Gaps in coverage lead to discarded reads or, worse, incorrect assemblies that mistakenly merge fragments from different transcripts. The principle is simple: garbage in, garbage out. Collectively, these five limitations explain why no amount of deeper sequencing, longer reads, or better software can rescue RNA-seq for TCR repertoire analysis. The problem is not insufficient data, it is that the essential information the contiguous V-CDR3-J linkage is destroyed during library preparation and cannot be reconstructed afterward. It is for these reasons that deeper sequencing, longer reads, or better bioinformatics tools will not be able to save RNA-Seq for TCR profiling [45, 46] (Fig. 2).

## Scope of RNA-seq technologies: beyond short-read platforms

The five fundamental limitations described above apply specifically to conventional short-read RNA-seq platforms, which are designed primarily for gene expression profiling and are by far the most widely used in public datasets [48]. However, it is important to acknowledge that other RNA-seq technologies particularly long-read and full-length RNA sequencing have been applied to TCR identification and repertoire analysis and may circumvent some of these limitations. Long-read RNA-seq platforms, such as PacBio Iso-Seq and Oxford Nanopore Technologies (ONT), can generate reads exceeding 10 kb, which is sufficient to span the entire V-CDR3-J region in a single read [44, 48]. This directly addresses the fragmentation and short-read limitations described above. Indeed, several studies have demonstrated successful TCR repertoire profiling using long-read RNA-seq, including full-length TCRα and TCRβ chain reconstruction and paired chain analysis when combined with single-cell partitioning [11]. These platforms also reduce 3′ bias, as they do not rely on fragmentation-based library preparation. However, long-read RNA-seq has its own limitations: higher per-base error rates (particularly for ONT), lower throughput, and higher cost compared to short-read platforms. More importantly, the vast majority of publicly available RNA-seq data including the >1 million samples in GEO and SRA were generated using short-read platforms [49]. Therefore, the retrospective mining strategy that motivates this review is primarily constrained by short-read data. We explicitly define the scope of this review as focusing on conventional short-read RNA-seq, while acknowledging that long-read technologies represent a promising complementary approach for prospective TCR profiling. We discuss long-read approaches further in the section on emerging technologies.

## TCR profiling beyond RNA-seq limits: AI and wet-lab innovations

While the limitations of RNA-seq are fundamental, the immunology community has not conceded defeat; instead, the field is converging on a pragmatic two-pronged strategy of computational enhancement to better mine existing data and experimental innovation to improve the quality of input data even from challenging samples, not about replacing TCR-seq but about expanding its reach and making TCR profiling feasible in previously inaccessible contexts [47, 50].

## Computational approaches: AI and machine learning

On the computational side, artificial intelligence (AI), particularly deep learning, offers a new frontier for tackling the assembly and ambiguity problems, with the core hypothesis being that a well-trained neural network can learn the statistical and biological rules of V(D)J recombination, allowing it to predict and correct the errors and ambiguities that plague traditional alignment tools [51]. However, a critical and unresolved question is whether AI can genuinely infer TCR sequences from gene expression profiles alone. The suggestion that AI may learn an "association between T cell gene expression profiles and the corresponding TCR repertoire" warrants careful scrutiny. The theoretical TCR sequence space is estimated at ~ 10^18 unique β chains, and the abundance distribution of each TCR further expands the repertoire state space [52]. In contrast, transcriptomic profiles comprise ~ 20,000 genes with continuous expression values. This represents a fundamental information asymmetry: the dimensionality of the repertoire space vastly exceeds that of the transcriptome [53]. While HLA restriction and antigen specificity may reduce the effective repertoire space in real samples, there is currently no evidence that gene expression profiles contain sufficient information to reconstruct TCR sequences de novo [54]. It is important to distinguish between different computational applications in this field. Some tools and studies focus on post-analysis of already-assembled repertoires [38], others provide benchmarking frameworks for evaluating TCR profiling methods, and yet others attempt direct CDR3 assembly from RNA-seq data [36]. While the latter demonstrates that CDR3 sequences can be computationally recovered from RNA-seq, the recovery rate remains low (4–6% of the infiltrating T cell repertoire), and the approach is limited by the fundamental constraints described above. However, these studies do not demonstrate reconstruction of actual TCR sequences from gene expression profiles alone. We therefore distinguish between two distinct categories: (a) AI-based error correction and assembly improvement, which is supported by existing evidence, and (b) AI-based imputation of missing TCR sequences from transcriptomes, which remains highly speculative and lacks empirical validation [55]. AI-based error correction and assembly improvement represent more tractable applications. Deep learning models can be trained to recognize patterns of true V-J recombination, such as the frequency of specific P- and N-nucleotide additions, and to filter out chimeric contigs that traditional assemblers generate, thereby scoring candidate CDR3 sequences based on biological plausibility and reducing false positives [55]. Tools like DeepTCR and ImmunoMap offer exciting possibilities for downstream analysis of TCR repertoires, but their application to de novo assembly from poor-quality RNA-seq data is still in the research phase. These approaches complement rather than replace traditional assembly pipelines [56]. Generative repertoire modeling using AI to design novel TCR sequences with desired specificity is a separate and promising field [38]. However, this is fundamentally different from inferring native TCR sequences from transcriptomic data. We emphasize this distinction to avoid overstating the potential of AI for RNA-seq-based repertoire mining.

## Wet-lab innovations

The most reliable solutions, in fact, are not computational but experimental, and several innovative techniques are expanding the boundaries of TCR profiling.

### Probe-based enrichment (hybridization capture)

This addresses the transcript abundance problem directly by using biotinylated RNA or DNA “baits” designed to hybridize to TCR constant regions and V-region families rather than relying on PCR amplification (which can be biased), thereby capturing TCR transcripts from a complex library of fragments and effectively enriching them by 100- to 1000-fold without amplification biases, making it possible to retrieve TCR sequences from bulk RNA-seq datasets including from FFPE tissues [57].

### FUME-TCRseq (full-length multiplex enhanced TCR-seq)

In this technique, the strength of 5′ RACE for retrieving full-length TCR transcripts is coupled with a multiplex PCR method involving just one highly efficient reaction for amplifying all V-J cassettes, overcoming the primer bias issues of traditional multiplex PCR and providing a highly sensitive method to recover full-length, paired alpha–beta TCRs from low-input samples such as fine-needle aspirates or small tumor biopsies [58].

### rhTCRseq (repertoire and HLA-matched TCR-seq)

This goes beyond mere TCR recovery by linking the TCR sequence to the peptide-HLA (pHLA) class of the individual, using specially engineered soluble HLA multimers containing a unique DNA barcode to physically “pull down” a T cell based on its antigen specificity and then sequence the matched TCR, allowing for the direct linkage of the TCR sequence to its cognate antigen, a holy grail of immunotherapy discovery [59].

### Long-read RNA-seq for TCR profiling

**Fig. 3:**
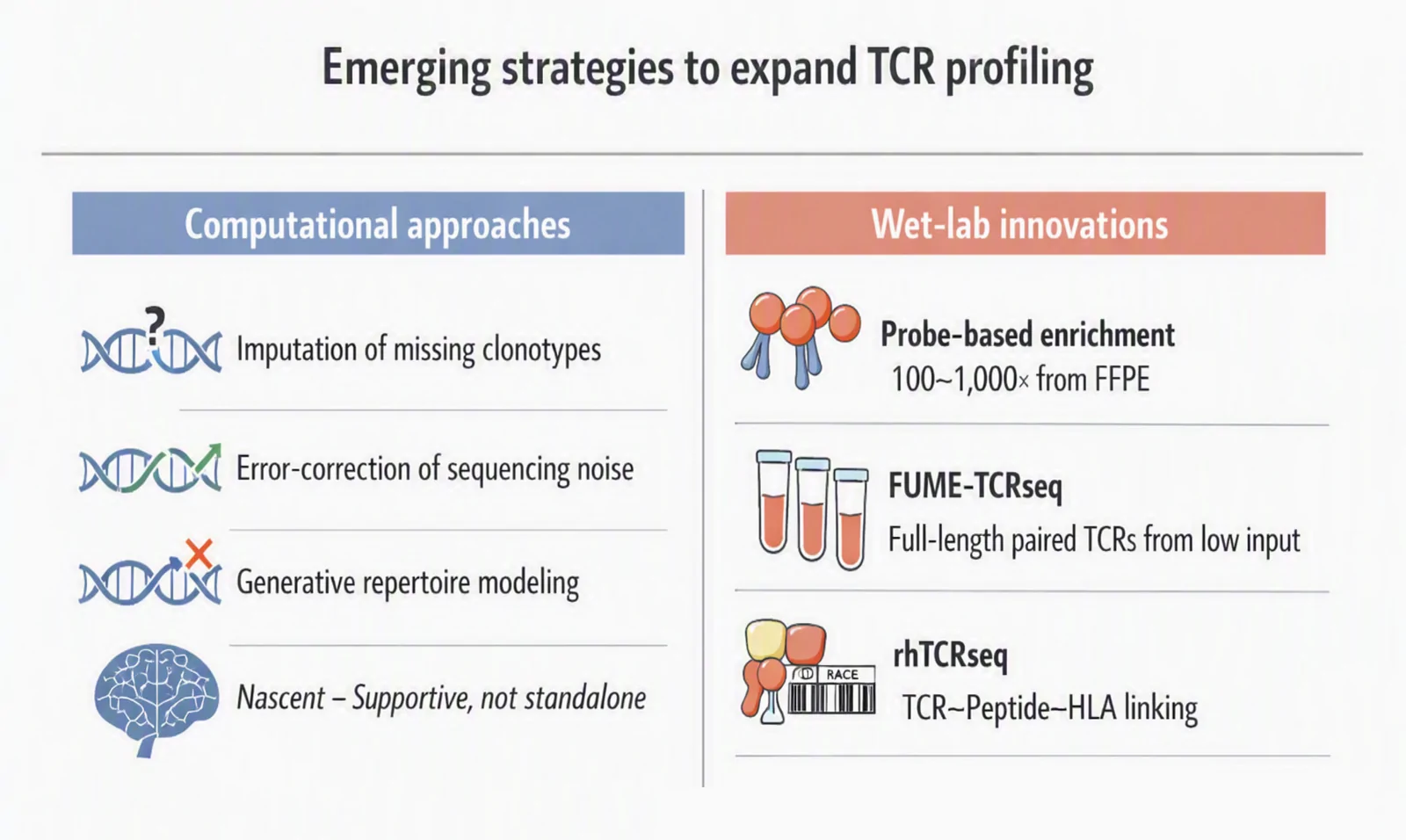
Emerging strategies to expand TCR profiling. Computational AI models offer error correction but remain nascent for de novo TCR inference; imputation from transcriptomes faces fundamental information-theoretic barriers. Wet-lab innovations include probe-based enrichment (from 100- to 1000-fold from FFPE), FUME-TCRseq (full-length paired TCRs from low-input samples), rhTCRseq (linking TCR to peptide-HLA specificity), and long-read RNA-seq (full-length TCR transcripts in single reads). These approaches augment, rather than replace, the gold standard TCR-seq

As discussed above, long-read platforms such as PacBio Iso-Seq and ONT can generate full-length TCR transcripts in single reads, overcoming fragmentation and short-read limitations [52]. When combined with single-cell barcoding, these approaches can achieve paired αβ chain recovery. However, their application to existing short-read datasets is not possible, and their adoption for large-scale repertoire studies remains limited by cost and throughput [44] (Fig. 3).

## Conclusion

The quest to characterize the T cell receptor repertoire is central to modern immunology, driving advances in cancer immunotherapy, vaccine development, and our understanding of autoimmune diseases [42]. The allure of repurposing the vast ocean of existing RNA-seq data for this purpose is a testament to our ingenuity; yet, as we have demonstrated, this path is paved with fundamental limitations [11]. The inherent scarcity of TCR transcripts, the constraints of short-read sequencing, and the ambiguities of hypervariable region assembly consistently prove that RNA-seq, as a standard technology, is ill-suited to replace dedicated TCR-seq. We have synthesized quantitative evidence from head-to-head benchmarking studies demonstrating that RNA-seq typically recovers only 3–5% of unique clonotypes, with poor concordance in frequency estimates, limited αβ pairing, and systematic underestimation of diversity metrics. These data substantiate the conceptual arguments and provide a clear picture of the magnitude of the gap. We have synthesized a wide body of evidence showing that computational tools like MiXCR and TRUST4, while exceptionally powerful for their intended purposes, hit a barrier of diminishing returns when applied to non-enriched data. The failures are not due to software flaws but to an irreconcilable mismatch between the biology of the TCR and the design of RNA-seq. As articulated in our five fundamental limitations, the problem is not insufficient data; it is that the essential information—the contiguous V-CDR3-J linkage—is destroyed during library preparation and cannot be reconstructed afterward. However, the story does not end in failure. The emergence of AI-driven computational strategies offers a glimmer of hope for better error correction and assembly improvement, potentially expanding the utility of existing datasets. We emphasize, however, that AI-based imputation of TCR sequences from transcriptomes faces fundamental information-theoretic barriers and currently lacks empirical support. The more realistic near-term applications of AI are in error correction and false-positive reduction, where deep learning can complement traditional assembly pipelines. More importantly, the development of innovative wet-lab techniques from hybridization capture to FUME-TCRseq and rhTCRseq demonstrates that we can overcome these limitations not by replacing the gold standard, but by augmenting it. These methods allow us to access TCR profiles from degraded specimens, low-input samples, and even to link specificity directly to sequence [28]. Long-read RNA-seq technologies represent a complementary approach that can overcome fragmentation and short-read limitations, though they are not applicable to retrospective mining of existing short-read datasets. The field’s future lies not in a single technology, but in a pragmatic integration of approaches. RNA-seq will remain invaluable for studying the transcriptomic state of T cells and the tumor microenvironment. TCR-seq, both bulk and single-cell, will continue to be the gold standard for full clonal resolution. The new wave of enrichment and functional techniques will unlock previously inaccessible clinical samples. Ultimately, the adaptive immune system’s complexity demands a multi-omic perspective. By recognizing the strengths and inherent limitations of each tool, we can build a more complete picture of the T cell repertoire and, in doing so, design more effective and personalized immunotherapies for cancer and beyond.

## Data Availability

No datasets were generated or analysed during the current study.
